# A Federated Approach for Adaptive Urban Sound Classification on TinyML Edge Devices

**DOI:** 10.3390/s26092854

**Published:** 2026-05-02

**Authors:** Athanasios Trigkas, Dimitrios Piromalis, Panagiotis Papageorgas

**Affiliations:** Department of Electrical and Electronics Engineering, University of West Attica, 12244 Athens, Greece; atrigkas@uniwa.gr (A.T.); ppapag@uniwa.gr (P.P.)

**Keywords:** federated learning, TinyML, urban sound classification, edge computing, smart sensors, smart cities, distributed sensor networks, on-device learning, privacy, acoustic monitoring

## Abstract

**Highlights:**

**What are the main findings?**

- A federated Tiny Machine Learning (TinyML) architecture with a trainable on-device classifier head enables real-time multiclass urban sound classification on microcontrollers, improving accuracy from 78.27% to ~85% under cross-dataset domain shift.
- Exchanging only a 231-parameter classifier head (~1.2 kB) via Message Queuing Telemetry Transport (MQTT) supports communication-efficient collaborative learning.

**What are the implications of the main findings?**

- Microcontroller-class edge devices can support adaptive real-time acoustic systems.
- Federated coordination can be achieved with minimal communication cost while preserving data locality.

**Abstract:**

Cities exhibit sound patterns that vary across locations and time, while transmitting raw audio introduces communication and privacy concerns. We present a federated TinyML architecture for real-time urban sound classification on microcontroller-class edge devices. A compact audio embedding network is deployed as a frozen feature extractor, while a lightweight classifier head is trained on-device and shared via MQTT, enabling communication-efficient collaborative learning. The system is evaluated on ESP32 (Espressif Systems, Shanghai, China) hardware under cross-dataset transfer from UrbanSound8K to SONYC. Domain shift reduces baseline accuracy from 90.39% to 78.27%, while local adaptation and federated aggregation improve accuracy to approximately 85%, recovering most of the performance loss. Repeated aggregation further improves macro-F1 and class balance across heterogeneous data. Embedded measurements confirm real-time inference (~250 ms per window) with negligible overhead, while each update exchanges only a compact classifier head (~1.2 kB). These results demonstrate that adaptive classification can be achieved on resource-constrained nodes in distributed smart-city networks.

## 1. Introduction

Cities are characterized by sounds that change over time due to infrastructure development, mobility trends, seasonal effects, and human activity. In addition, they also vary across different parts of a city, depending on traffic, socioeconomic conditions, and the built environment [1,2]. Monitoring these soundscapes is becoming an integral part of smart city systems, with applications in environmental noise, public safety, and well-being in crowded areas [1,3,4]. Automatic urban sound classification is therefore an emerging technology aligning with the United Nations Sustainable Development Goal 11 (Sustainable Cities and Communities) and Goal 9 (Industry, Innovation and Infrastructure).

Nowadays, systems rely heavily on cloud-based data collection and model training. While this practice can be effective under controlled settings, it can also present limitations in real-world deployments. Transmitting raw audio introduces communication overhead and latency, and raises privacy concerns [5]. Moreover, these models often struggle to generalize across heterogeneous environments, where acoustic characteristics differ across locations and constantly evolve.

Urban sensing systems are often participatory or opportunistic, where heterogeneous devices join and leave over time [6]. Moreover, many federated learning approaches assume regular client availability. In real-world deployments, device connectivity and participation vary, and this can bias training toward nodes that are consistently online [7].

TinyML offers a promising alternative by enabling audio processing directly on low-power embedded devices. Local inference reduces communication requirements and limits the exposure of sensitive data, aligning with privacy requirements for acoustic sensing [5]. However, most TinyML-based systems remain inference-only, relying on models trained offline and deployed unchanged to edge devices [8,9]. This approach limits adaptability in changing urban environments.

Federated learning (FL) is a training approach in which multiple devices collaborate to optimize a shared model by exchanging model parameters [10]. FL is attractive for edge-based sensing scenarios, as it enables collaborative learning while preserving data locality. However, recent surveys highlight that applying FL to TinyML devices remains challenging due to computational, memory, and communication constraints, and that few works demonstrate complete on-device learning pipelines under realistic conditions [8,11,12].

**Figure 1:**
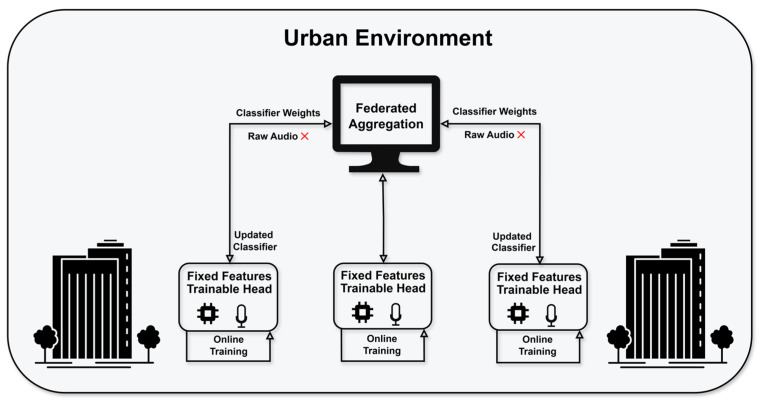
Federated TinyML architecture overview. Edge nodes perform local inference, while aggregation exchanges only classifier parameters. Arrows indicate parameter exchange, red ‘X’ denotes that raw audio is not transmitted, icons represent local training and audio input.

We propose a federated TinyML system for urban sound classification (Figure 1). A frozen audio embedding network provides stable features, while a lightweight classifier head is trained on-device, enabling adaptive learning without retraining the full neural network.

Adaptation is evaluated under domain shift using a cross-dataset setup where the embedding network is trained on UrbanSound8K [13], and evaluated on the SONYC dataset [14], which differs in recording conditions and annotation structure. Earlier work suggests that cross-domain evaluation provides a more realistic assessment of model robustness than training and testing on a single dataset [15,16,17], particularly for TinyML systems [18]. Consistent with this perspective, the experimental evaluation assesses static, locally adaptive, and federated deployments under dataset shift to reflect urban sound sensing domains.

FL enables collaboration among nodes by exchanging only classifier-head parameters and minimal metadata, keeping communication cost low and preserving data locality. Federated aggregation is evaluated using five ESP32 nodes communicating via MQTT-based coordination, demonstrating collaborative learning under embedded deployment. The main contributions of this work are summarized below:

1. An end-to-end federated TinyML architecture for real-time multiclass urban sound classification.
2. An efficient federated coordination framework based on asynchronous MQTT communication, enabling collaboration across dynamically participating edge nodes.
3. A comprehensive evaluation under realistic domain shift, including cross-dataset transfer, simulation-based analysis of heterogeneous data distributions, and the impact of client scaling on federated performance.
4. Benchmarking of the considered ESP32 devices to evaluate inference latency, memory footprint, and communication cost of the proposed system.

## 2. Related Work

This section reviews technical work related to federated and on-device learning for TinyML systems, as well as TinyML-based urban audio classification for smart city applications, focusing on implemented systems and experimental studies.

### 2.1. Federated and On-Device Learning for TinyML Systems

FL has been explored in TinyML contexts by constraining model size and limiting the scope of on-device training to operate within microcontroller-level memory and compute budgets [12]. TinyFedTL presents a federated transfer learning approach based on a split-model design, where a frozen feature extractor is combined with a lightweight classifier head, enabling collaborative learning across constrained devices [19]. The approach relies on repeated federated rounds in which only the classifier parameters are exchanged and aggregated. In the reported implementation, model weights and biases are communicated between the device and the server via a wired serial interface.

FL has also been combined with meta-learning techniques for constrained devices. TinyReptile applies federated meta-learning by updating a shared model initialization rather than aggregating trained model parameters. Each device performs a small number of local gradient steps on the full network, and the server updates the initialization so that future devices can adapt from this starting point [18]. The paper discusses serial and batched client coordination modes for communicating updates to the server, but does not specify a concrete communication protocol. In this setting, the learned initialization is not intended to represent a stable task-specific model.

A recent study addresses system-level aspects of FL on constrained Internet of Things (IoT) devices, focusing on communication efficiency, energy consumption, and privacy preservation during federated execution [20]. The study evaluates FL using small multilayer perceptron models (MLP) on low-dimensional sensor data in a gateway-based IoT architecture, where edge nodes transmit updates to a central gateway using low-power long-range communication. The work concentrates on reducing the overhead of federated coordination through techniques such as compression, quantization, and communication-aware aggregation.

Federated on-device learning has also been demonstrated in application-agnostic IoT settings using shallow neural networks or small convolutional models for sensing and regression tasks [9]. Due to their limited representational capacity, such models are typically applied to low-dimensional sensor data and tasks with relatively simple decision boundaries. Similarly, audio-specific FL has been explored for keyword spotting using small MLP models [21]. This demonstrates feasibility under resource constraints, but still relies on simple architectures, limiting applicability to small vocabularies and requiring wired communication for model updates.

In addition to federated approaches, local on-device learning without collaboration has been studied. TinyOL investigates online learning mechanisms for streaming data on TinyML devices by attaching a trainable layer to a frozen neural network and updating it incrementally on-device [22]. Another work demonstrates that full convolutional neural network (CNN) training is feasible on microcontroller-class devices under strict memory constraints through specialized training pipelines and memory-aware backpropagation [23]. This represents an interesting proof of concept for on-device learning, but is limited to single-device training.

### 2.2. TinyML-Based Urban Audio Classification for Smart City Applications

TinyML techniques have been applied to urban audio analysis for smart city applications, focusing on low-power and embedded deployment of sound monitoring systems. Infrastructure-free urban noise monitoring systems demonstrate that convolutional audio embeddings can be computed efficiently on low-power sensor nodes and deployed in real-world city settings for long-term monitoring [24], with inference results transmitted over low-power wide-area networks (LPWANs).

A TinyML-based pipeline for urban noise source classification on embedded and edge devices is presented in [25], targeting applications such as environmental monitoring and urban noise management. Other work focuses on the design and optimization of CNN architectures for urban sound detection, addressing memory and computational constraints [26].

Embedding-centric approaches have also been explored for urban audio classification. Student–teacher and approximation techniques are applied to generate compact, domain-specific audio embeddings suitable for deployment on constrained edge devices [27]. Beyond supervised classification, unsupervised TinyML approaches have been proposed for detecting anomalies in urban noise patterns using autoencoder-based models [28].

Earlier work evaluating classical machine learning techniques for urban sound recognition on embedded systems provides reference results on execution time and accuracy before the adoption of deep learning-based TinyML [29].

### 2.3. Relation to This Work

The proposed system adopts a head-only on-device training approach in which a lightweight classifier is trained locally on top of a frozen feature extractor, following approaches demonstrated in TinyOL and extended to federated settings in TinyFedTL. These works demonstrate the feasibility of incremental learning and federated transfer learning under strict memory and computation constraints.

However, existing studies typically evaluate simplified learning scenarios and communication setups. For instance, TinyFedTL focuses on binary image classification tasks and performs model exchange through serial communication with a central host, while other microcontroller FL prototypes rely on tightly controlled experimental environments and small vocabulary recognition tasks [21]. As a result, the interaction between distributed TinyML sensory nodes and IoT communication is not extensively explored. Furthermore, prior work generally evaluates training and inference within a single dataset or task domain.

In this work, the head-only training approach is applied to urban sound classification using a CNN-based federated TinyML architecture. The system introduces MQTT-based coordination to support asynchronous communication between edge nodes and evaluates model adaptation under cross-dataset shift. The use of a fixed embedding is motivated by the memory, computational, and energy constraints of microcontroller-class devices, as it avoids the overhead associated with backpropagation through the full network while still enabling adaptation through a lightweight classifier head.

Direct comparison with prior federated TinyML studies is limited by differences in tasks, datasets, hardware platforms, and communication settings. For reference, TinyFedTL is evaluated on Arduino Nano 33 BLE Sense (Arduino SA, Chiasso, Switzerland) devices, reporting approximately 210 kB memory usage and communication payloads exceeding 6 kB per update, with transfer times on the order of tens of seconds over serial communication. TinyReptile is evaluated on Raspberry Pi 4 (Raspberry Pi Foundation, Cambridge, UK) and Arduino Nano 33 BLE Sense platforms and reports memory requirements ranging from a few kilobytes to several hundred kilobytes, depending on the task. TinyOL is evaluated on Arduino Nano 33 BLE Sense devices and reports model performance, without detailed computational measurements. In this work, the system is evaluated on ESP32 devices, with inference latency of approximately 250 ms per window, inference-related memory usage of about 50 kB static RAM, and communication payloads of approximately 1.2 kB per update.

## 3. System Architecture and Methodology

The proposed system follows a split-model design in which a CNN-based audio embedding network is trained offline and deployed as a frozen feature extractor using TensorFlow Lite Micro (TFLM) (Google LLC, Mountain View, CA, USA). Local on-device learning is applied to a lightweight linear classifier head operating on the embedding output, which is trained incrementally using stochastic gradient descent (SGD) updates [19,20].

FL is applied only to the classifier head, which is periodically transmitted along with minimal metadata to a coordinating server via MQTT, where it is aggregated and redistributed. The system supports serial and pulse density modulation (PDM) audio input and uses mel-frequency cepstral coefficients (MFCC) for feature extraction. Table 1 summarizes the key configuration parameters of the proposed system.

### 3.1. Edge Node Architecture and On-Device Learning

**Figure 2:**
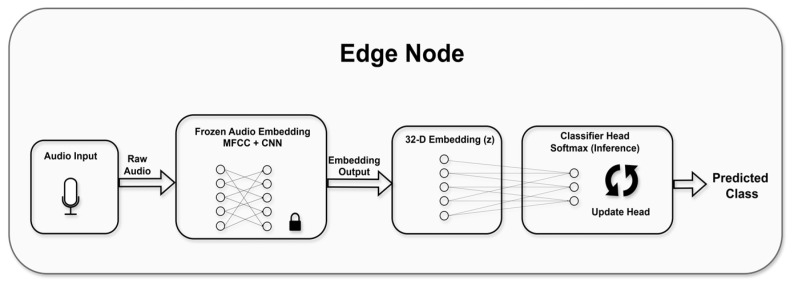
Edge-node processing pipeline. Audio is transformed into a 32-D feature vector by a frozen embedding network, then classified by a lightweight trainable head with on-device updates. Arrows show data flow, lines and circles represent network connections and symbols indicate processing.

Figure 2 illustrates the internal architecture of a single sensing node and the split-model learning pipeline deployed at the edge, in which each node performs audio acquisition, feature extraction, inference, and classifier updates locally.

#### 3.1.1. Acquisition and Feature Extraction

Audio is acquired at a sampling rate of 16 kHz and processed using continuous overlapping 2 s windows. Predictions are generated every 0.5 s using sliding-window inference while maintaining real-time sound classification along with low latency and computational cost.

Each window is processed by the frozen embedding, which extracts MFCC features and maps them to a 32-dimensional embedding using a compact CNN trained offline and deployed with the Edge Impulse (EdgeImpulse Inc., San Jose, CA, USA) TinyML toolchain [30]. The detailed configuration of the signal-processing pipeline and window parameters is described in Section 4.4.

#### 3.1.2. Classifier Head Architecture

For each window, the frozen embedding model outputs a feature vector $z\in R^{D}$, with D=32 corresponding to the embedding dimension. Classification is performed by a lightweight linear classifier head parameterized by a weight matrix $W\in R^{C\times D}$ and a bias vector $b\in R^{C}$, where C is the number of target sound classes. Class scores are computed as

(1) s=Wz+b,

where $s\in R^{C}$ is the vector of unnormalized class scores, and $s_{c}$ is the score associated with the class c. These scores are then transformed into class probabilities using the softmax function,

(2) $$p_{c}=\frac{e^{s_{c}}}{\sum_{j=1}^{C}e^{s_{j}}},\;c=1,\;\ldots ,C,$$

where $p_{c}$ is the predicted probability of class c and e is the base of the natural logarithm. The predicted sound class corresponds to the index of the maximum probability output.

The classifier head is initialized from the final layer of the offline-trained model. It is implemented directly in C/C++ using linear and softmax operations, allowing local training without embedded training runtimes or backpropagation through the neural network.

#### 3.1.3. On-Device Training

The classifier head is trained incrementally using locally available labeled samples. For each input segment, the frozen embedding network produces a fixed-dimensional feature vector, which is combined with the corresponding ground-truth label to update the classifier parameters using a single SGD step based on the predicted class probabilities defined in Section 3.1.2. Updates are applied immediately and independently for each segment, without storing previous samples or accumulating across multiple inputs.

The learning rate controls the magnitude of the parameter update applied after each labeled segment. Because a single SGD step is performed per segment, it determines how strongly each new sample influences the classifier. Larger values enable faster adaptation but result in larger parameter shifts, while smaller values provide more stable updates. The learning rate is configurable in the firmware and can be adjusted at runtime.

A local counter records the number of update steps performed by each device. It is implemented as a 32-bit unsigned integer, which allows more than $4\times 10^{9}$ updates before overflow. This value is used during federated aggregation to weight client contributions according to the amount of local training performed.

### 3.2. Federated Learning and Coordination

**Figure 3:**
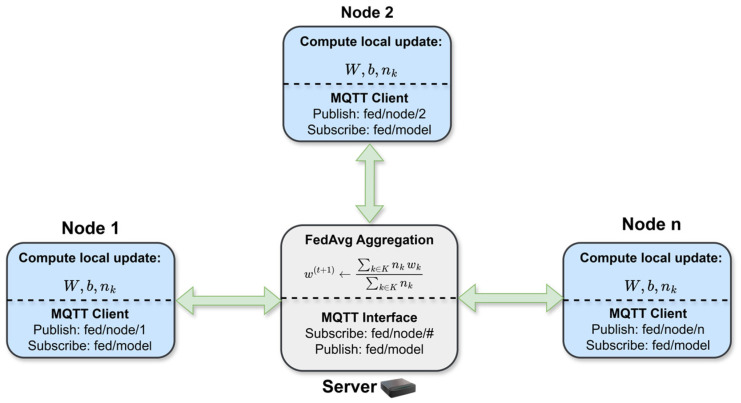
Asynchronous federated coordination across edge nodes. Nodes publish classifier-head updates via MQTT, aggregated with Federated Averaging (FedAvg) and redistributed as a global model. Arrows show communication flow, dashed lines separate local and communication stages.

Collaboration among distributed nodes is achieved by exchanging only the classifier head parameters while keeping raw audio and intermediate features local, as depicted in Figure 3. In order to coordinate nodes and the central aggregator, we use the MQTT publish–subscribe protocol. Each update message contains a tiny payload with the classifier weight matrix, bias vector, and the number of local update steps associated with the model.

Following this scheme, nodes upload updates with minimal communication cost and whenever connectivity is available, while the server aggregates received models before republishing them. Two topics are used for model exchange:

- fed/node/<id> is the uplink topic where each node publishes its local classifier head, identified by its ID.
- fed/model is the broadcast topic that is used by the server to distribute the aggregated classifier head.

#### 3.2.1. Boot-Time Synchronization

Upon boot or reconnection, each node subscribes to the “fed/model” topic and begins inference using its current classifier head. When a new global model is published, the node replaces its local parameters and continues on-device learning from the synchronized state. Local classifier heads are periodically uploaded via “fed/node/<id>”, enabling asynchronous participation under intermittent connectivity.

#### 3.2.2. Server-Side Aggregation

At the server, received classifier heads are aggregated using weighted averaging based on Federated Averaging (FedAvg) [10]. Let K represent the set of nodes whose updates are included in a given aggregation step, and let $w_{k}$ be the head parameters received from the node k. The server computes an aggregated global head $w^{\left(t+1\right)}$ as a weighted average of the received heads:

(3) $$w^{\left(t+1\right)}=\frac{\sum_{k\in K}n_{k}w_{k}}{\sum_{j\in K}n_{j}},$$

where $n_{k}$ represents the number of local update steps contributing to the update. The resulting global head is broadcast to all subscribed nodes via the same “fed/model” topic.

## 4. Experimental Setup

This section presents the setup used to evaluate the proposed federated TinyML system. Experiments follow the split-model design introduced in Section 3, with a frozen embedding network on each node and a lightweight classifier head updated locally and shared through federated aggregation.

### 4.1. Embedded Hardware Platform

Experiments were conducted using ESP32 boards as representative microcontroller-class edge devices for TinyML-based urban sound sensing. The ESP32 features a Tensilica Xtensa LX6 32-bit processor, which operates at 240 MHz, 520 kB of on-chip SRAM, and 4 MB of external SPI flash memory. The platform also provides optimized digital signal processing through the ESP-NN library, which can be utilized by TFLM to accelerate convolutional operations during inference.

The ESP32, presented in Table 2, was selected because it offers a practical balance between computational capability, memory resources, and cost while remaining representative of constrained IoT sensing nodes. Although more lightweight constrained platforms exist, the audio embedding network used in this work is relatively heavier than those used in TinyML keyword-spotting tasks. The ESP32, therefore, enables reliable real-time inference while still reflecting realistic deployment conditions for distributed acoustic sensors.

Connectivity and runtime support also influenced this choice. The ESP32 integrates Wi-Fi for direct communication with the aggregation server and provides native support for FreeRTOS and the ESP-IDF “esp-mqtt” client (version 5.5.1) used for MQTT communication. Together, these components support concurrent streaming inference, efficient neural network execution, and federated model communication on the device.

ESP32 development modules are widely available at low cost (typically $5–$10 USD), making them suitable for large-scale deployments of distributed acoustic sensing nodes in urban environments.

### 4.2. Dataset Preparation

The audio embedding model used in all experiments is trained offline using the UrbanSound8K dataset. Because evaluation is conducted on SONYC as the target domain and only a subset of labels overlaps between the two datasets, training and evaluation are restricted to a selected seven-class subset: “car_horn”, “children_playing”, “dog_bark”, “engine_idling”, “jackhammer”, “siren”, and “street_music”.

UrbanSound8K consists of short audio clips with a single dominant sound event, which makes it well-suited for training compact models for TinyML. In embedded inference, models typically operate on short windows and classify one main sound event at a time under limited resources. The dataset’s event-focused structure and short clip duration, therefore, match the requirements of microcontroller-based systems.

The UrbanSound8K class “children_playing” is aligned with the broader human-voice supercategory in SONYC (talking, shouting, large-crowd). Although SONYC does not provide a child-specific label, both categories represent human vocal activity in public urban environments. In the dataset, “children_playing” is characterized by vocal expressions such as talking, shouting, and group interactions. This class is therefore interpreted as a generalized human vocal activity category, and this alignment preserves information about human activity, which is important for urban sound monitoring.

UrbanSound8K originally contains ten sound classes. The classes “air_conditioner”, “gun_shot”, and “drilling” are excluded from this study because they do not have reliable counterparts in the SONYC taxonomy. In particular, SONYC’s related category to drilling, “rock-drill”, differs acoustically from the electric drilling recordings in UrbanSound8K. Restricting the labels to the overlapping subset helps ensure that cross-dataset comparisons primarily reflect differences between datasets rather than mismatches in class definitions. Taxonomy mismatch is a known challenge in cross-domain learning [31], and excluding classes without reliable counterparts helps avoid negative transfer, since source-domain outlier classes can interfere with feature transfer across shared classes [32].

Prior work has addressed taxonomy mismatch by merging several UrbanSound8K classes, such as “engine_idling”, “jackhammer”, “drilling”, and “street_music”, into a broader “background” or “ambient noise” category for deployment-specific applications [33]. While this grouping can simplify classification and improve stability, it may mask confusion between acoustically similar classes under domain shift. We therefore preserve the original class distinctions within the selected subset.

We adopt the predefined UrbanSound8K folds to preserve the standard dataset partitioning. However, since evaluation is performed using the SONYC dataset, all folds were used for training instead of reserving fold 10 as a test set. The resulting dataset contains 6358 audio clips, and the class distribution is summarized in Table 3.

### 4.3. SONYC Mapping and Federated Partitioning

To construct the target-domain dataset for cross-dataset evaluation and federated experiments, the SONYC dataset is used. Unlike UrbanSound8K, SONYC provides multi-label annotations with a hierarchical taxonomy and proximity metadata, which differ from the single-label structure of UrbanSound8K [34]. Deterministic filtering and alignment procedures are therefore applied to obtain a subset compatible with the previously defined 7-class taxonomy.

#### 4.3.1. Label Alignment to UrbanSound8K

SONYC categories were mapped to the subset of UrbanSound8K classes selected for this study. SONYC provides multi-label annotations with hierarchical categories and annotator voting.

We considered a class to be present when it received at least two positive votes from citizen annotators, following the annotation strategy described in the SONYC dataset documentation. To further reduce noise and ambiguity, the following constraints were applied:

Citizen annotators only: only annotations from citizen contributors were used.

Proximity filtering: clips labeled as far were discarded to prioritize events that match the recordings in UrbanSound8K.

Single-label constraint for non-music classes: for all classes except music, clips were retained only when exactly one matching class met the required vote threshold.

Engine restriction: for the “engine_idling” class, only small and medium-sounding engines were kept. This reduces overlap with heavy machinery and better matches typical idling vehicle sounds.

Music annotations in SONYC require special handling because the dataset frequently contains multi-label scenes where music co-occurs with other urban sound sources (e.g., traffic or crowd noise). To align with the UrbanSound8K “street_music” class, all SONYC music-related subtypes were merged into a single category. Clips were retained only when music constituted the dominant supercategory. This constraint removes clips where music is only a faint component while still preserving a sufficient number of samples. The resulting aligned dataset contains 2986 clips distributed across the seven target classes, as summarized in Table 4.

#### 4.3.2. Federated-Style Partitioning

For federated experiments, the resulting SONYC dataset is partitioned into five client subsets and a shared evaluation set (Table 5). The evaluation set is sampled per class and removed before distributing the remaining 2029 clips across the five clients. This results in approximately IID (Independent and Identically Distributed) client distributions, while we explore non-IID scenarios separately through simulation experiments described later. To account for partition variability, this process is repeated using three different random seeds and no clip appears in more than one subset.

### 4.4. Impulse Creation

In Edge Impulse, we configure an impulse to process audio sampled at 16 kHz using fixed windows of 2 s duration with a 0.5 s stride, producing a new prediction every 0.5 s during continuous inference. MFCC features were extracted with a frame length of 25 ms, frame stride of 20 ms, 32 Mel filters, a 512-point FFT, a normalization window size of 151, frequency range from 80 Hz to the Nyquist frequency, and a pre-emphasis coefficient of 0.98. MFCCs were selected due to their effectiveness for capturing complex acoustic characteristics in urban sound classification on embedded IoT devices, where MFCC-based CNN models have shown strong performance [3,29].

The number of cepstral coefficients was increased empirically to 20, relative to the default value of 13, based on Edge Impulse autotuning. Increasing the number of coefficients allowed the model to retain additional spectral detail for heterogeneous sound events while remaining within the memory and runtime constraints of hardware. This design choice aligns with earlier work showing that MFCC-based features can capture complex acoustic characteristics in urban sound environments [2].

Alternative techniques were also explored during the impulse design stage. In particular, Mel-frequency energy (MFE) features were tested as a lightweight alternative to MFCCs. Experiments showed that MFE-based models achieved similar validation accuracy while resulting in slightly faster inference and reduced computation. However, inspection of class predictions revealed weaker separability for acoustically similar classes, specifically “children_playing” and “street_music”. These classes contain overlapping background sounds common in urban environments, and MFCC features provided more stable discrimination between them. For this reason, MFCC features were retained as the final representation for the experiments reported.

### 4.5. Model Training

**Figure 4:**
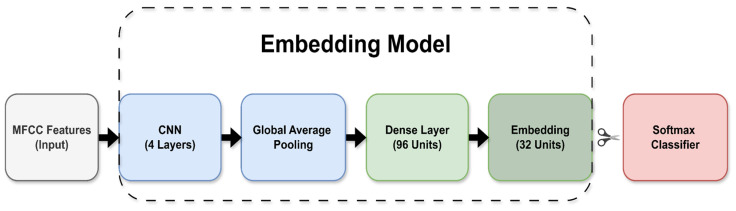
Audio embedding network architecture. The dashed region denotes the deployed embedding, with the final softmax replaced by a lightweight on-device classifier head. Arrows indicate data flow; colors distinguish model components.

The audio embedding network consists of a lightweight CNN operating on MFCC features, as illustrated in Figure 4. The architecture includes four convolutional layers, each followed by batch normalization and Rectified Linear Unit (ReLU) activation, with max-pooling applied after the first two convolutional layers to reduce the time resolution. These layers are followed by global average pooling and two fully connected layers, where the first dense layer contains 96 units and the second produces a 32-dimensional embedding representation that serves as the input to the lightweight classifier head used in this work.

During training, augmentation and regularization mechanisms are applied to reduce overfitting. Gaussian noise with a standard deviation of 0.10 is added to the input features, while SpecAugment time masking randomly hides short temporal segments of the input feature representation during training. Dropout is applied throughout the network after convolutional blocks and dense layers, and L2 weight regularization is applied to the dense layer preceding the embedding layer. The network was trained for 100 epochs using categorical cross-entropy loss and the Adam optimizer. We set the initial learning rate to 0.001 and the batch size to 32.

Various architectural and training configurations were explored. These included deeper convolutional configurations, alternative convolutional formulations using Conv2D and Conv3D layers, and different regularization settings. While some configurations produced small improvements in validation accuracy, they generally increased inference time or model complexity. The final architecture and training configuration were therefore selected to balance predictive performance with the computational constraints of microcontroller-class edge devices.

### 4.6. Model Post-Processing

Edge Impulse exported the trained network as a full end-to-end Keras model, including both feature extraction and classification layers. For embedded deployment, the default exported classifier model was replaced with a sliced version of the same trained network, retaining the 32-dimensional intermediate layer as the embedding output, following a similar approach to [19,22]. This post-processing step does not modify learned parameters and preserves the original network topology up to the selected embedding layer.

To support deployment on microcontroller-class hardware, the embedding model was exported in both 32-bit Floating Point (FP32) and fully quantized 8-bit Integer (INT8) TFLM formats using the Edge Impulse toolchain. INT8 post-training quantization was performed using MFCC training data exported from Edge Impulse, ensuring that calibration reflects the same feature distribution seen during model training. This allows appropriate activation ranges to be determined for quantized inference and results in stable embedding behavior during streaming execution on the ESP32. The embedding network remains frozen throughout all experiments and is never updated or exchanged between devices, serving solely as a consistent feature extractor for the on-device classifier head described in Section 3.2.

### 4.7. Embedded Node Implementation

Each edge node was implemented on an ESP32 microcontroller using PlatformIO (version 6.1.18) and TFLM. To expose the embedding representation for on-device learning, the autogenerated impulse metadata was modified so that the model output corresponds to the 32-dimensional embedding layer rather than the original softmax classifier. The impulse configuration was updated to define 32 placeholder output categories, allowing the embedding vector to be returned through the standard inference interface. The original classifier layer parameters were extracted separately and used to initialize the classifier head.

Minor adjustments were made to the TFLM runtime to allow deployment on ESP32. Initial experiments using dynamically allocated tensor arenas occasionally failed due to heap fragmentation, and the tensor arena was therefore allocated in static DRAM. The interpreter was configured with a static “AllOpsResolver”, ensuring deterministic memory allocation and operator availability during inference. Communication with the coordinating server is performed using the ESP-IDF “esp-mqtt” client library.

### 4.8. Audio Streaming-Based Training and Evaluation Protocol

**Figure 5:**
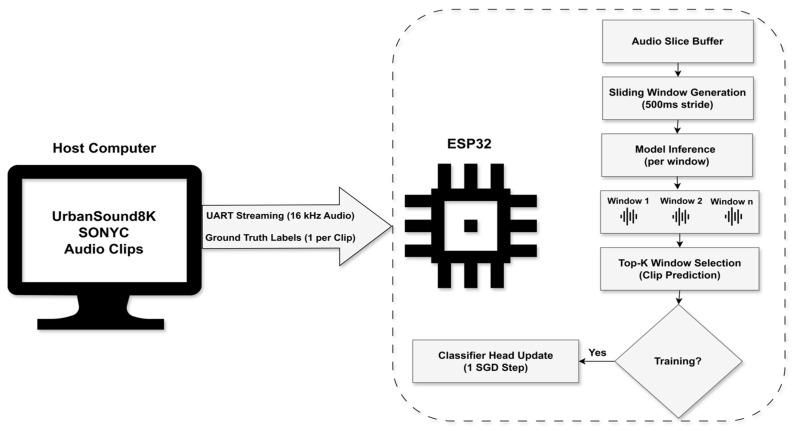
HIL evaluation and training pipeline. The host streams audio to the ESP32, which processes it using sliding-window inference (500 ms stride, 20 windows per 10 s clip), and aggregates the window into a clip-level prediction. Each labeled clip triggers a classifier-head update. Arrows indicate data and decision flow, dashed region denotes ESP32 processing.

To ensure reproducible evaluation under realistic embedded conditions, both training and evaluation experiments are performed using a hardware-in-the-loop (HIL) pipeline, as illustrated in Figure 5. Pre-recorded audio clips from the UrbanSound8K and SONYC datasets are replayed as WAV files on a host computer and streamed to the ESP32 over a high-speed universal asynchronous receiver–transmitter (UART) connection.

All audio files are resampled to a sampling rate of 16 kHz and transmitted to the device as sequential audio slices. The ESP32 buffers incoming samples and processes them using the Edge Impulse continuous inference API, which applies the same window and stride configuration defined in Section 4.4. As a result, multiple inference windows are generated for each audio clip.

Because multiple windows are produced for a single clip, window-level outputs must be aggregated to obtain a stable clip-level prediction. The windows with the highest confidence are selected and averaged to produce a single representative embedding for the clip. This aggregation strategy follows a similar approach to [35] for converting frame-level predictions into clip-level decisions.

The same streaming pipeline is used for both training and evaluation. During training, the ground-truth class label for each streamed clip is transmitted to the device and used to update the classifier head. During evaluation, the same inference pipeline is executed without updating the classifier parameters.

In addition to supervised adaptation, preliminary experiments were conducted using unsupervised and semi-supervised update strategies based on pseudo-labeling and confidence-thresholded predictions, including variants inspired by [36]. However, under the cross-dataset domain shift between UrbanSound8K and SONYC, pseudo-label errors occasionally reinforced incorrect predictions and resulted in unstable behavior. Since these approaches did not improve performance, the evaluation in this work focuses on supervised updates.

### 4.9. Server-Side Coordination and Aggregation

We implement server-side coordination using lightweight MQTT messaging, whereas message routing is provided by an Eclipse Mosquitto broker (Eclipse Foundation, Brussels, Belgium, version 2.0.14) running on the server host. The server subscribes to node-originated update topics and publishes aggregated classifier-head parameters to participating nodes. The classifier head consists of 928 bytes in binary and is encoded in base64 before MQTT transmission, which results in a payload of ~1.2 kB per update. Model uploads and broadcasts use Quality of Service (QoS 1) to improve robustness under uncertain Wi-Fi connectivity.

Aggregation is performed using weighted averaging, as described in Section 3.2.2, and updates are processed asynchronously without synchronized training rounds. The server is implemented in Python (version 3.13) and supports checkpointing of local and aggregated classifier heads as NumPy archive files for offline analysis. It can request heads on demand and rebroadcast aggregated models to resynchronize participating devices.

## 5. Experimental Evaluation and Embedded Performance

This section evaluates the proposed federated TinyML system under the cross-dataset setting described in Section 4. Performance is measured using overall accuracy, macro-F1, and weighted-F1. Earlier work shows that performance can vary across datasets and class distributions, making a single metric insufficient [15,16]. In FL, models must also perform across different client environments [17]. Therefore, accuracy provides a general measure of performance, while macro-F1 captures per-class behavior, and weighted-F1 reflects performance under realistic class distributions.

All experiments are conducted using continuous streaming inference with overlapping windows, and sound events do not align to clip boundaries, reflecting deployment in which sensors operate continuously. The window duration and stride are fixed and match the configuration described in Section 3.1.1. Experiments are repeated across three random seeds, and results are reported as the mean ± standard deviation. In addition to experiments conducted on ESP32 devices, simulation-based federated experiments are presented in Section 5.7 to evaluate data heterogeneity and client scalability.

### 5.1. Embedded Inference Performance

In addition to classification accuracy, TinyML deployments must satisfy strict constraints on latency and memory. This section evaluates the embedded inference performance of the proposed system on the ESP32 platform, including inference latency, the impact of ESP-NN acceleration, and memory usage. Because the sensing pipeline performs streaming inference with a 500 ms stride between windows, the system must execute substantially faster than this interval to maintain real-time operation. The following measurements confirm that the deployed model operates within these constraints while remaining compatible with the limited resources of hardware.

#### 5.1.1. Inference Latency and ESP-NN Acceleration

Inference latency was evaluated on the ESP32 using the continuous streaming configuration described in Section 4.4 and real audio input captured from a digital PDM microphone (Adafruit PDM MEMS microphone, Adafruit Industries, New York, NY, USA) through the ESP32 I2S peripheral.

To ensure robust continuous acquisition, audio capture, and inference are executed as separate FreeRTOS tasks pinned to different ESP32 CPU cores. The capture task continuously records microphone samples and stores them in a ring buffer, while the inference task processes completed windows as they become available. This separation prevents inference from blocking audio sampling and ensures that incoming audio is not dropped even if processing latency exceeds the stride interval.

Table 6 summarizes the measured latency across different deployment configurations. We report two metrics: the capture interval and the total inference latency. The first represents the effective time between consecutive inference windows processed by the system and reflects how frequently new audio data is analyzed during continuous streaming; under ideal real-time conditions, it matches the configured 500 ms stride. However, if inference becomes slower than the capture rate, the system continues buffering incoming audio, causing the capture interval to increase as processing falls behind. The total inference latency corresponds to the time required to complete feature extraction, run the neural network inference, and evaluate the trainable classifier head.

The results show that the INT8 deployment with ESP-NN acceleration sustains real-time streaming at the configured stride. The total inference latency is ~250 ms per window, meaning that inference completes twice as fast as the required interval. Consequently, the system maintains real-time operation while retaining significant capacity for additional tasks or more complex models.

Disabling ESP-NN significantly increases INT8 inference latency to approximately 1.26 s per window, causing the processing task to fall behind the capture rate. In this case, the effective interval between processed windows increases to approximately 1.5 s as the system processes buffered audio. For FP32 inference, latency exceeds these constraints regardless of whether ESP-NN is enabled, with execution times around 1.7 s per window. This suggests that floating-point inference on the ESP32 is unsuitable for real-time streaming.

For reference, Edge Impulse Studio reports estimated execution times of approximately 843 ms for the INT8 model and 1252 ms for the FP32 model, including both feature extraction and classification. While these values are lower than the latencies measured in our deployment, they provide a useful approximation of the computational cost and correctly reflect the relative difference between quantized and floating-point inference. In particular, the Edge Impulse estimates indicate that FP32 inference requires approximately 500 ms more computation than the INT8 model, which is consistent with the difference observed in our experiments with ESP-NN disabled.

Across all configurations, the additional classifier head used for on-device adaptation introduces negligible overhead (~0.18 ms), confirming that the proposed mechanism does not practically affect inference latency.

#### 5.1.2. Memory and Flash Footprint

Memory usage was evaluated on the ESP32 using an inference-only configuration that excludes Wi-Fi and MQTT components to isolate model execution costs. Static RAM and flash usage were obtained from the PlatformIO linker report, and heap statistics were measured on-device after continuous streaming inference. Results are summarized in Table 7.

In the INT8 deployment, the tensor arena reservation is 20,147 bytes, closely matching Edge Impulse’s estimation of 20.0 kB peak RAM. The full firmware uses 49,968 bytes of static RAM, and the minimum free heap after continuous inference results in 177,424 bytes, which indicates substantial available memory. Regarding flash memory, the size used is 724,469 bytes.

The tensor arena reservation increases to 51,635 bytes for the FP32 deployment, and Edge Impulse similarly estimates 50.8 kB peak RAM. Static RAM increases to 81,456 bytes while the minimum free heap decreases to 146,004 bytes. In a similar fashion, the footprint in flash increases to 917,453 bytes.

Edge Impulse reports model flash sizes of 124.0 kB for INT8 and 284.4 kB for the FP32 model, corresponding to an approximate 160 kB difference between INT8 and FP32 models. The measured flash difference on the ESP32 is 192,984 bytes, which is similarly consistent in magnitude.

The classifier head consists of 231 fp32 parameters (7 × 32 weights and 7 biases), corresponding to 924 bytes. This component is identical in both INT8 and FP32 deployments and contributes negligibly to memory.

### 5.2. Embedding Validation on Embedded Hardware

To evaluate numerical consistency across execution environments, the frozen embedding model is assessed using the same UrbanSound8K dataset as in offline training. All audio clips, ranging from approximately 2 to 4 s in duration, are processed using continuous streaming inference. Clip-level predictions are obtained using the UART connection described in Section 4.8. Because this experiment focuses strictly on numerical consistency rather than generalization, no adaptation, retraining, or federated aggregation is performed.

Starting with Edge Impulse, we observe that the FP32 deployment achieves 91.30% accuracy, 91.34% weighted-F1, and 90.40% macro-F1. The INT8 deployment reaches similar values, with 91.34% accuracy, 91.40% weighted-F1, and 90.36% macro-F1, indicating negligible performance loss due to post-training quantization.

**Figure 6:**
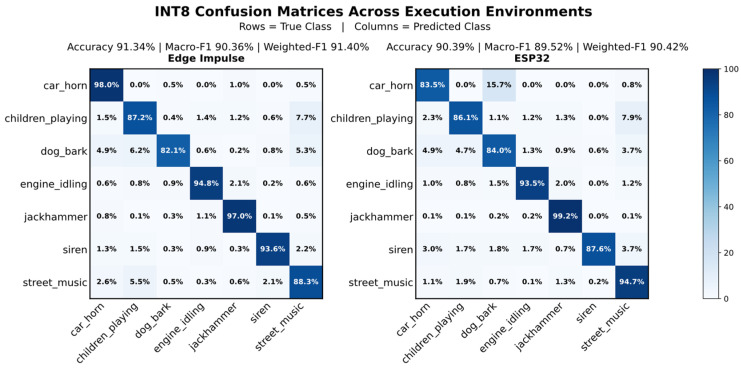
Row-normalized confusion matrices (%) for INT8 inference on Edge Impulse and ESP32. Consistent diagonal dominance indicates preserved class separability after quantization.

We then evaluate the embedding on the ESP32, where the INT8 version slightly outperforms FP32, achieving 90.39% accuracy (90.42% weighted-F1, 89.52% macro-F1), while FP32 remains about one percentage point lower. Across both environments, the deviation relative to Edge Impulse stays within approximately two percentage points. These differences are mainly related to continuous streaming and the constraints of embedded execution. Table 8 summarizes performance across the Edge Impulse evaluation and ESP32, while Figure 6 provides a class-wise view of INT8 prediction behavior.

At the class level, performance is consistent across execution environments, as shown by the row-normalized confusion matrices for the INT8 deployment. High recall is observed for “jackhammer” (97.0%) and “engine_idling” (94.8%), with strong performance also for “siren” (93.6%) and “street_music” (88.3%). These classes retain comparably high recall on ESP32, while classes characterized by shorter transient events, such as “car_horn” and “siren”, show moderate reductions. Regardless of these shifts, no class shows collapse or instability, and class performance is preserved across both environments.

### 5.3. Cross-Dataset Evaluation Before On-Device Adaptation

After validating embedding consistency across execution environments, we evaluate cross-dataset generalization under domain shift using the SONYC dataset. The embedding network and classifier head trained on UrbanSound8K are deployed on the ESP32 without any on-device learning or federated aggregation. Pre-recorded SONYC audio clips (10 s duration) are streamed to the ESP32 over the same UART connection.

Under zero-adaptation, the ESP32 deployment achieves 78.27% accuracy, 75.63% macro-F1, and 79.28% weighted-F1 over the SONYC evaluation set (957 clips). Compared to UrbanSound8K performance (90–91% accuracy in Section 5.2), this represents a decrease of approximately 12–13%, reflecting the data and class differences between the datasets.

Performance varies across classes. Strong results are observed for “siren”, “dog_bark”, and “children_playing”, which show that the model works well for sound categories with distinctive acoustic patterns. Notably, mapping “children_playing” to broader human vocal activity in SONYC shows that the model can still recognize similar sounds even when the labels do not match exactly across datasets.

**Figure 7:**
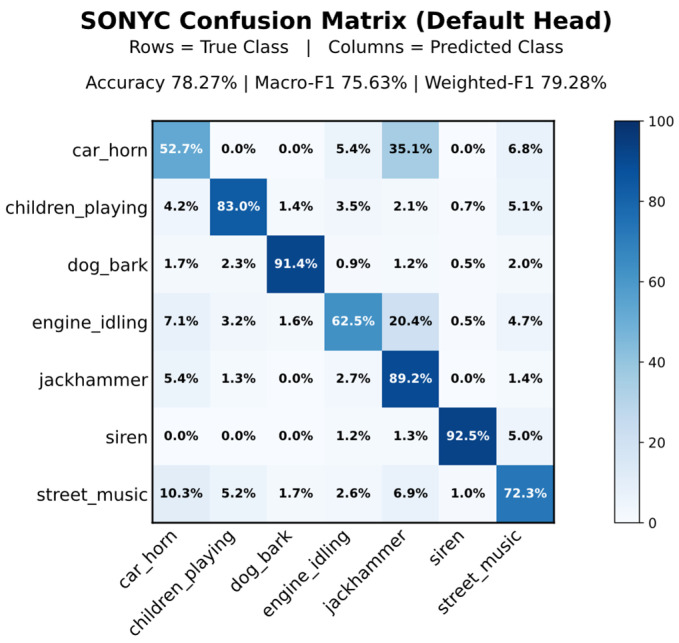
Row-normalized confusion matrix (%) for SONYC cross-dataset evaluation on ESP32. Shows class performance and cross-domain confusion under dataset shift.

In contrast, we observe reduced performance for classes such as “engine_idling” and “car_horn”, while “street_music” and “jackhammer” demonstrate greater confusion, as these sounds can differ significantly across clips and can often overlap with other categories. As illustrated in Figure 7, the dominant diagonal structure is preserved, but misclassifications between classes increase, reflecting cross-dataset confusion. Although all classes experience degradation relative to UrbanSound8K, no class collapses entirely.

### 5.4. Local Adaptation and Federated Initialization

Following the zero-adaptation evaluation in Section 5.3, we next examine whether individual edge nodes can locally adapt to the target domain. Using the same HIL streaming procedure, each ESP32 device trains for one full local epoch using its corresponding SONYC partition described in Section 4.3.2. The classifier head is updated online with SGD using a learning rate of 0.001. Table 9 summarizes performance before and after adaptation.

Compared to the zero-adaptation baseline of 78.27% accuracy, one epoch of local training improves accuracy by approximately three to five percentage points, resulting in a range from approximately 80.4% to 82.9% across devices, showing consistent performance across nodes. For macro-F1, we observe an increase of around two to four percentage points, which indicates that the gains are distributed across classes.

At this stage, each node adapts independently using its own data, resulting in personalized classifier heads that reflect the characteristics of the corresponding partition. To evaluate collaborative learning, the five locally adapted classifier heads are aggregated using a single round of FedAvg, and the resulting federated model achieves 82.80 ± 0.24% accuracy and 79.12 ± 0.38% macro-F1, placing it within the same performance range as the individually adapted devices. Notably, the federated model achieves a high macro-F1 compared to the evaluated models, indicating improved class balance.

Class-level trends remain broadly consistent with those detected before adaptation. Strong classes such as “dog_bark” and “siren” remain stable, while weaker ones show mixed behaviour, with “engine_idling” showing consistent improvements and “street_music” remaining comparatively challenging and not exhibiting improvement.

Overall, these results demonstrate that local on-device adaptation can effectively reduce cross-dataset shift, while lightweight federated aggregation can further improve class balance without hurting overall performance. This observation motivates the multi-round federated learning experiments presented in the following sections.

### 5.5. Multi-Round Federated Training on ESP32

We next evaluate how the frequency of federated aggregation affects adaptation performance. Using the SONYC client partitions defined in Section 4.3.2, each device trains the classifier head on its local partition using SGD with a learning rate of 0.001, similar to the local adaptation setting described in Section 5.4. We compare three small-scale communication schedules that maintain the same total local training budget per device but differ in how often federated aggregation occurs:

Frequent, with six federated rounds and one local epoch per round.

Baseline, with three federated rounds and two epochs per round.

Sparse, with a single federated round after six local epochs.

**Figure 8:**
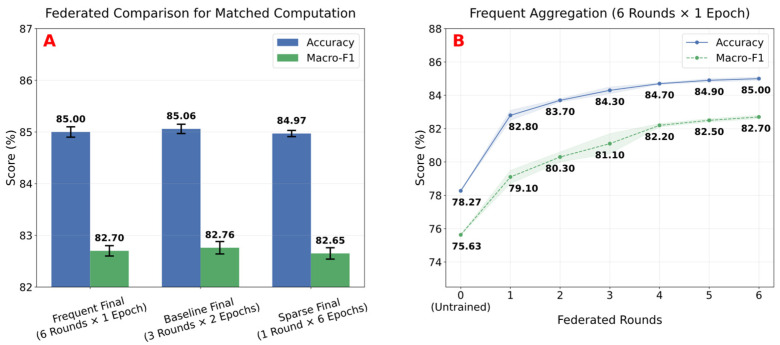
Federated training schedules under matched compute budgets (mean ± standard deviation across three seeds). (**A**) Final accuracy and macro-F1 for Frequent, Baseline, and Sparse settings (six local epochs per device). (**B**) Accuracy and macro-F1 across rounds for the Frequent setting, shaded areas indicate ± standard deviation.

All three schedules achieve similar final accuracy, indicating that federated adaptation remains effective even when communication is reduced, as illustrated in Figure 8. The Frequent schedule reaches 85.00 ± 0.10% accuracy and 82.70 ± 0.10% macro-F1 after six rounds, while the Baseline schedule achieves 85.06 ± 0.09% accuracy and 82.76 ± 0.12% macro-F1, and the Sparse schedule achieves 84.97 ± 0.06% accuracy and 82.65 ± 0.11% macro-F1 after a single federated round.

Since all schedules perform the same total amount of local training (six epochs per device), the small spread in final accuracy indicates that such a training budget can recover sufficient performance drop across datasets, regardless of how often aggregation is performed. In particular, the final Baseline model reaches similar accuracy to the Frequent schedule, suggesting that aggregation frequency has a limited impact on final accuracy in this experiment. However, the slightly higher macro-F1 values observed with repeated federation indicate that more rounds can potentially improve class balance across clients.

We also observe this behavior in the round-by-round development of the Frequent configuration shown in Figure 8. Starting from the unadapted model, performance improves rapidly in the first rounds and then gradually stabilizes. After six rounds, the model has moved from 78.27% to 85.00 ± 0.10% accuracy and from 75.63% to 82.70 ± 0.10% macro-F1.

**Figure 9:**
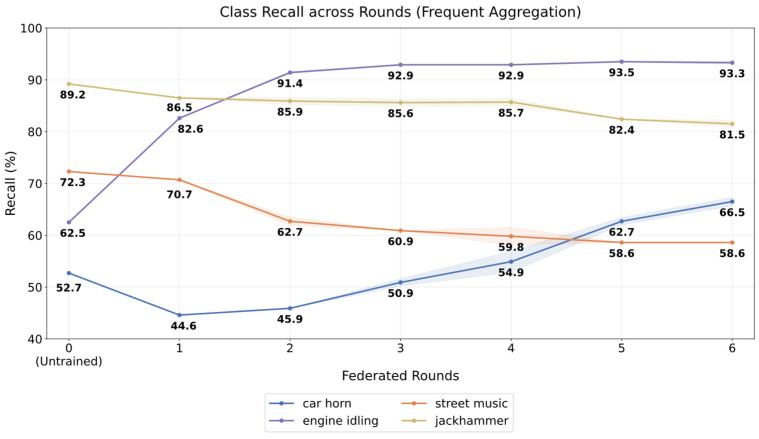
Class-wise recall across rounds for the Frequent configuration. Lines show mean recall across three seeds, and shaded regions denote standard ± deviation.

The class-level effect of the Frequent configuration is shown in Figure 9, which illustrates the evolution of class recall across rounds for a subset of representative classes. As adaptation progresses, distinct trends can be observed across categories. Recall improves substantially for “engine_idling”, increasing from 62.5% to 93.3 ± 0.3%, while “car_horn” shows a more gradual improvement from 52.7% to 66.5 ± 0.8%, whereas the “street_music” class experiences a reduction from 72.3% to 58.6 ± 0.0%, likely due to greater heterogeneity in the dataset, with small decreases also observed in classes that are already well separated, such as “jackhammer”, which decreases from 89.2% to 81.5 ± 0.7%.

Overall, the ESP32 federated results show that lightweight on-device learning can reduce differences across datasets despite device constraints. The matched-compute comparison further suggests that more frequent federation may influence class-level behaviour, although its impact on final performance remains limited.

To evaluate how adaptation to the SONYC target domain influences the original training domain, the final Baseline model was also evaluated on the UrbanSound8K dataset. After training on SONYC, the model achieves 88.37% accuracy, 87.25% macro-F1, and 88.51% weighted-F1 on UrbanSound8K. Compared with the performance reported in Section 5.2, this corresponds to a reduction of approximately two percentage points. This indicates that head-only federated adaptation substantially improves target-domain performance while preserving most of the structure learned from the original dataset.

### 5.6. MQTT Communication Overhead

Communication overhead was measured from runtime logs produced by the server using the Paho MQTT client (Eclipse Foundation, Brussels, Belgium, version 2.1.0). Each ESP32 node transmits only the classifier head parameters, resulting in a compact message payload. In the current implementation, the binary model parameters are base64-encoded before transmission, producing an MQTT message of approximately 1.2 kB per client update, as summarized in Table 10.

Timing measurements were also recorded during federation rounds to capture head collection, aggregation, and model distribution delays. The head collection window corresponds to the time allowed for nodes to respond to a broadcast head request. Model update latency represents the end-to-end delay between publishing the aggregated head and the ESP32 device reporting that the new federated head has been applied.

### 5.7. Simulation-Based Federated Experiments

To extend the ESP32 experiments, additional federated learning experiments are conducted in simulation using a Python implementation on a Linux machine, where the same Edge Impulse embedding model is reused through a compiled shared library (.so) based on the Edge Impulse POSIX port, ensuring that feature extraction remains consistent with the ESP32 firmware.

In this setup, the 2029 SONYC clips used for client partitions are treated as a common training pool. The classifier head is initialized using the same warm-start parameters as in the ESP32 and updated using the same training procedure, with all computations performed locally on the same machine without UART-based streaming. Federated aggregation is also performed locally without network communication.

The simulation follows the ESP32 baseline configuration, with a learning rate of 0.001 and two local epochs per communication round. To account for variability, we repeat each configuration with three different random seeds.

#### 5.7.1. Data Heterogeneity

We first consider a five-client setting and evaluate the effect of data heterogeneity. The shared training pool is partitioned using both IID sampling and non-IID Dirichlet splits with α = 1.0, 0.5, and 0.1, a commonly used approach for simulating varying degrees of client data heterogeneity in federated learning [37]. In particular, smaller values of α correspond to more skewed client distributions, while larger values yield more balanced partitions, with α = 0.1 and α = 0.5 widely used to represent high and moderate heterogeneity, respectively [38]. Federated training is performed for 30 communication rounds with 2 local epochs for each client, reflecting the baseline schedule of ESP32.

**Figure 10:**
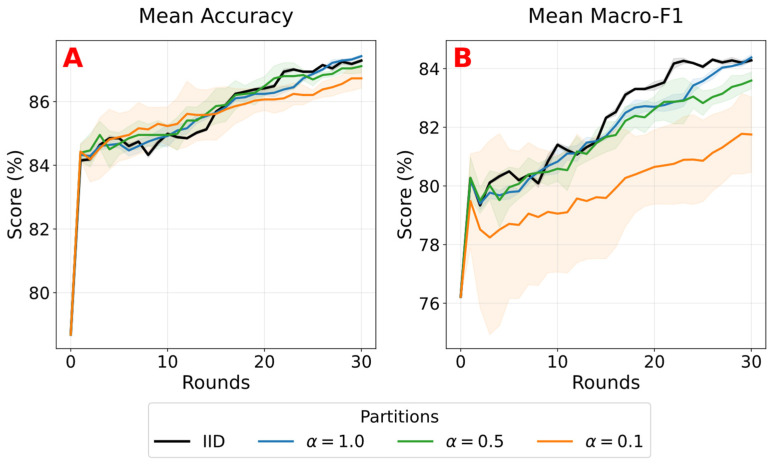
Mean accuracy (**A**) and macro-F1 (**B**) across federated rounds for different Dirichlet partitions. Curves show the mean over three seeds, with shaded regions indicating ± standard deviation at each round.

Figure 10 shows the evolution of mean accuracy across federated rounds for the different data partitioning strategies. Similar accuracy levels are observed for all configurations, with IID reaching 87.29% ± 0.05% and α = 0.1 reaching 86.73% ± 0.31% at round 30, indicating limited sensitivity to data heterogeneity. The effect is more evident in macro-F1, where IID achieves 84.28% ± 0.06%, while α = 0.1 drops to 81.75% ± 1.29% and higher variability across seeds is exhibited. This seems most pronounced during early rounds, where α = 0.1 shows a standard deviation of 3.31% at round 3 compared to 0.05% under IID. This variability decreases over time, but a performance gap persists, indicating that non-IID data primarily affects class balance rather than overall accuracy.

#### 5.7.2. Client Scalability

**Figure 11:**
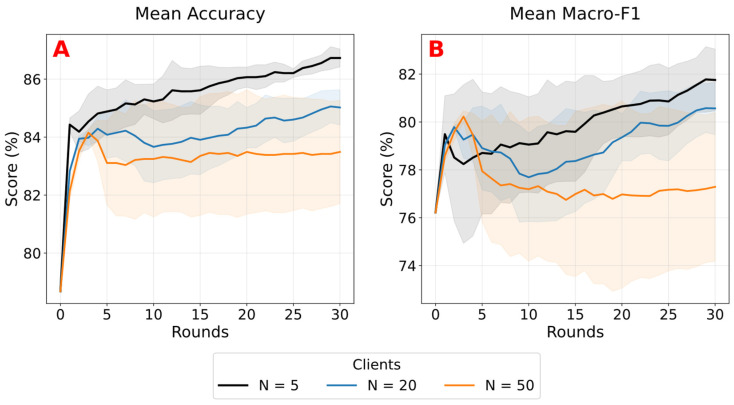
Mean accuracy (**A**) and macro-F1 (**B**) across federated rounds for different client counts (N = 5, 20, 50) under a strongly non-IID partition (α = 0.1). Curves show the mean across three seeds, with shaded regions indicating ± standard deviation at each round.

To examine scalability, we vary the number of clients while keeping the total dataset size fixed, evaluating configurations with 5, 20, and 50 clients under strongly non-IID partitions (α = 0.1) for 30 communication rounds. As shown in Figure 11, increasing the number of clients leads to slower convergence and reduced final performance. At round 30, we notice a decrease in accuracy, from 86.73% ± 0.31% for 5 clients to 85.02% ± 0.61% for 20 clients and 83.49% ± 1.78% for 50 clients, with a similar trend observed for macro-F1, which drops from 81.75% ± 1.29% to 80.57% ± 1.09% and 77.29% ± 3.11%, respectively. This degradation is accompanied by a substantial increase in variability, particularly for macro-F1, indicating less stable training as the number of clients increases.

#### 5.7.3. Class-Wise Analysis

To further analyze the impact of heterogeneity and scaling, we examine class-wise recall across federated training. While global metrics such as accuracy and macro-F1 provide an overall view, they can hide class-specific behavior.

Table 11 reports recall for all classes at initialization (round 0) and after 30 communication rounds. The results reveal substantial variation across classes, with “street_music” exhibiting the largest degradation, decreasing from 87.90% to 55.20% under IID and further to 27.60% ± 9.76% under α = 0.1 with 50 clients. This class also shows high variability across seeds, indicating strong sensitivity to both class imbalance and reduced data availability. On the other hand, “car_horn” shows consistent improvement during training, increasing from 52.70% to 83.80% under IID and further to 86.90% ± 2.55% under α = 0.1 with 5 clients. However, this improvement diminishes as the number of clients increases, dropping to 58.10% ± 13.40% for 50 clients.

Other classes exhibit more stable behavior, with “children_playing” maintaining high recall across all configurations, improving from 82.80% to above 91% in all cases. Similarly, “dog_bark” remains largely unaffected, with recall consistently around 90–93%. “siren” shows only minor degradation from 92.50% to 88.70% under IID and remains relatively stable under non-IID settings. In contrast, “jackhammer” shows moderate degradation under non-IID conditions, decreasing from 89.20% to 79.70% under IID and further to 69.83% ± 2.80% for 50 clients, suggesting that certain classes are more sensitive to uneven data distributions.

Overall, these results indicate that the impact of non-IID data and client count is strongly class-dependent. Classes with distinctive acoustic signatures remain robust, while more heterogeneous categories are highly affected. This effect is partly influenced by dataset composition. For example, “children_playing”, which has the largest number of samples in the SONYC dataset (Table 5), consistently achieves high recall across all configurations. In contrast, “street_music”, which has fewer samples, shows performance degradation and variability. However, sample count alone does not fully explain the observed behavior, as acoustically distinctive classes such as “dog_bark” and “siren” remain stable despite having fewer samples.

### 5.8. Full-Model Upper Bound

Following the split-model experiments, we implement a full-model federated learning upper bound in Python to establish a reference for performance. This setting removes the constraints of TinyML deployment by allowing all model parameters to be updated during training, while preserving the same data, feature representation, and federated setup used in the ESP32 experiments.

The implementation uses the same MFCC feature extraction pipeline from Edge Impulse, compiled as a shared library (.so), along with the original Keras model that was used for the ESP32 deployment. Model inference and training are performed using the TensorFlow Lite Python API (Google LLC, Mountain View, CA, USA, version 2.20.0) with an FP32 model. Unlike typical machine learning pipelines, MFCC features are computed through the Edge Impulse pipeline, as in the embedded implementation.

Training is performed at the clip level, following the same logic as on the ESP32. Batches of 32 clips are used during training, and optimization is performed with Adam using a learning rate of 0.001, consistent with both the original model training and the embedded experiments. This setup allows for all model parameters to be trained, and federated aggregation is applied over the full model. To examine the behavior of this setting, we use the same fixed five-client partitioning as in the ESP32 experiment, repeated across three seeds. Federated training is performed for three communication rounds with two local epochs per round, matching the baseline communication schedule.

Table 12 summarizes the performance metrics for the two experiments, along with selected key classes. Overall, the full-model setting achieves higher final performance compared to the ESP32 implementation. Starting from an initial accuracy of 79.62% and 77.89% macro-F1 at Round 0, the model reaches 88.78% ± 0.27% accuracy and 86.63% ± 0.18% macro-F1 after three rounds, corresponding to an improvement of approximately 9 percentage points and outperforming the head-only setting by around 3–4 percentage points. The full-model setting improves performance while leading to more stable class-wise behavior. Although the gain in accuracy is moderate, the results indicate a more consistent distribution of performance across classes.

In particular, substantial differences are observed for the “street_music” class, as performance decreases in both cases after training, but the decline is more pronounced under head-only adaptation, whereas in the full-model setting it remains closer to the Round 0 level (78.7% ± 1.6). A similar pattern is observed for “engine_idling”, where performance improves in both cases, but the ESP32 setting shows a stronger increase while the full-model setting leads to a more moderate change. At the same time, “car_horn” shows a strong improvement in the full-model setting, reaching high recall after training. The remaining classes, including “dog_bark”, “siren”, and “children_playing”, show more consistent behavior across both approaches.

## 6. Discussion

The results show that the proposed architecture can adapt to domain shift using only a lightweight linear classifier head trained directly on-device. This suggests that a stable embedding representation can support post-deployment adaptation through minimal parameter updates, which is particularly advantageous for resource-constrained embedded devices.

The cross-dataset evaluation illustrates the relevance of this mechanism under varying deployment conditions. The datasets differ in recording pipelines, annotation procedures, and acoustic environments, reflecting the variability encountered across urban sensing locations. While the evaluation is limited to five clients and a single cross-dataset setting, it captures key aspects of domain shift in embedded urban sensing scenarios. The observed performance drop before adaptation and the recovery achieved through local and federated updates highlight the importance of post-deployment learning in dynamic environments.

Federated aggregation further improves stability across distributed nodes. While local training adapts each model to its client data, repeated aggregation helps redistribute decision boundaries across classes and improves class balance when clients observe heterogeneous data distributions.

The simulation results indicate that the proposed approach remains robust under non-IID data distributions, with only a limited impact on overall accuracy, although increasing the number of clients appears to slow convergence and lead to a substantial drop in performance. These effects suggest that non-IID data and larger client populations may disproportionately affect certain classes.

The comparison with the full-model upper-bound suggests a trade-off between efficiency and performance. While full-model training achieves higher accuracy and more consistent class-wise behavior, the overall improvement remains relatively modest, making the head-only approach a practical option for resource-constrained deployments due to its significantly lower computational and communication requirements.

A closer examination of individual classes provides further insight into these effects across both simulation and embedded settings. Classes such as “dog_bark” and “children_playing” remain consistently strong, while “siren” shows only minor degradation, indicating relative robustness to domain shift and data heterogeneity. In contrast, “street_music” exhibits notable performance degradation under head-only adaptation, while being better preserved in the full-model setting, suggesting increased confusion for this class when only the classifier head is updated. “car_horn” starts with relatively low performance but improves substantially after training, likely because its short and distinctive acoustic events can be captured within individual inference windows and reinforced during adaptation. On the other hand, “engine_idling” shows strong improvement under head-only adaptation, while the full-model setting leads to more moderate gains, which may be related to its more homogeneous acoustic structure, together with its relatively larger number of samples in the dataset. These observations suggest that class-level performance depends not only on data distribution but also on the acoustic characteristics of the sound events, and that more complex adaptation mechanisms may perform better for less distinctive classes.

The embedded measurements also confirm that the architecture appears well-suited for microcontroller deployments. The classifier head introduces negligible memory overhead, while quantized accelerated inference enables continuous streaming operation within the limits of the ESP32 platform. Together with a small communication payload, these characteristics may suggest practical deployment in distributed sensing networks with constrained bandwidth and device resources.

The compact parameter exchange may also facilitate deployment in LPWANs commonly used in urban sensing systems, such as LoRaWAN or NB-IoT. Because each federated update contains only the classifier-head parameters, the communication requirements remain small. Smaller payloads may also reduce the energy required for wireless transmission on battery-powered sensors, although a detailed evaluation of power consumption and energy efficiency was not performed.

### Challenges and Future Research

Energy consumption represents a primary consideration for practical deployment on battery-powered edge devices. Although the proposed system is designed to be lightweight in terms of computation and communication, energy usage was not explicitly measured in this study. In particular, energy consumption per inference and per communication round was not quantified. A detailed evaluation of power consumption and its relationship to communication frequency, as well as on-device training, remains an important direction for future work.

The evaluation on embedded hardware is limited to a small number of ESP32 clients and a single cross-dataset setting, which may restrict generalizability. While simulation experiments extend the analysis to larger client populations and non-IID data distributions, real-world scalability and communication dynamics remain to be further investigated in future work.

In this study, adaptation relied on manually labeled samples to evaluate performance, which may limit applicability in deployments where labeled data is not readily available. In practical smart-city scenarios, supervision could instead be obtained indirectly through sensors such as traffic counters, radar detectors, or motion sensors that confirm acoustic events. These signals could enable incremental classifier updates and support autonomous long-term adaptation. We use a FedAvg-based aggregation scheme, while alternative federated optimization methods, such as FedProx [39] or SCAFFOLD [40], which are designed to improve robustness under heterogeneous data distributions, are left for future work.

Although self-training was not realized, the proposed update mechanism can be compatible with a semi-supervised approach. Self-training methods iteratively retrain models using high-confidence pseudo-labels [41], and approaches such as FixMatch demonstrate that pseudo-labeling can effectively leverage unlabeled data [36]. Because our method updates only a compact linear head while keeping the shared embedding fixed, it limits parameter drift compared to full-network training and may support more stable distributed adaptation.

## 7. Conclusions

This work presented a federated TinyML architecture for urban sound classification on microcontroller-class edge nodes, designed to remain accurate under heterogeneous and evolving urban sound conditions without centralizing raw audio. A frozen embedding network provides stable features, while a lightweight linear softmax classifier head is trained on-device and shared via MQTT as compact parameter updates. The key results are:

- Generalization gap: Cross-dataset transfer from UrbanSound8K to SONYC reduces accuracy from 90.39% to 78.27%, highlighting the impact of domain shift.
- On-device adaptation: Local training increases accuracy to about 82% (78% macro-F1), while federated aggregation across five nodes further increases performance to about 85% accuracy (83% macro-F1).
- Real-time embedded feasibility: INT8 deployment achieves ~250 ms inference latency per window, enabling continuous inference with a 500 ms stride on ESP32.
- Minimal communication overhead: Each update exchanges only the 231-parameter classifier head, resulting in a payload of ~1.2 kB.
- Scalability and heterogeneity effects: Performance remains stable under non-IID conditions, but is influenced by the number of participating devices, with larger client populations reducing final accuracy.
- Full-model upper-bound comparison: In the full-model setting, we observe slightly higher accuracy, which appears to result from a more homogeneous class distribution compared to the head-only approach.

## Figures and Tables

**Table 1:** System Configuration and Design Parameters.

Category	Parameter	Value
Hardware	Target device	ESP32
Audio input	Input interface	Serial, optional PDM
Audio	Sampling and windowing	16 kHz, 2 s window
Digital Signal Processing (DSP)	Feature extraction	MFCC
Inference	Runtime	TFLM
Embedding	Model and dimension	CNN, 32
Classifier	Model and training	Linear softmax, on-device SGD
Federation	Shared parameters	Classifier head only
Communication	Protocol	MQTT

**Table 2:** Selected Platform Features.

Property	Specification
CPU	Tensilica Xtensa LX6, 32-bit
CPU clock speed	Up to 240 MHz
On-chip memory	520 kB SRAM
Flash storage	4 MB external SPI flash
Real-time operating system	FreeRTOS
MQTT client	ESP-IDF esp-mqtt
Neural network acceleration	ESP-NN
Development board cost	~$5–$10 USD

**Table 3:** UrbanSound8K Subset for Training the Embedding Model.

Class	Clips
car_horn	429
children_playing	1000
dog_bark	1000
engine_idling	1000
jackhammer	1000
siren	929
street_music	1000
Total	6358

**Table 4:** Label Alignment between SONYC Taxonomy and the UrbanSound8K 7-Class Subset.

Final Class	UrbanSound8K Label	SONYC Categories
engine_idling	engine_idling	engine: small-sounding-engine, medium-sounding-engine
jackhammer	jackhammer	machinery-impact: jackhammer
car_horn	car_horn	alert-signal: car-horn
siren	Siren	alert-signal: siren
dog_bark	dog_bark	dog: barking/whining
children_playing	children_playing	human-voice: talking, shouting, large-crowd
street_music	street_music	music: stationary, mobile, ice-cream-truck, uncertain-source (dominant)

**Table 5:** Distribution of SONYC Clips Across Federated Clients and Evaluation Test Set.

Class	Total	Evaluation	C1	C2	C3	C4	C5
car_horn	298	74	45	45	45	45	44
children_playing	929	429	100	100	100	100	100
dog_bark	232	58	35	35	35	35	34
engine_idling	684	184	100	100	100	100	100
jackhammer	294	74	44	44	44	44	44
siren	318	80	48	48	48	47	47
street_music	231	58	35	35	35	34	34
Total	2986	957	407	407	407	405	403

**Table 6:** Inference and Audio Capture Latency on ESP32 under Different Configurations.

Model Type	ESP-NN	Audio Capture Interval (ms)	Total Inference Latency (ms)	Head Prediction (ms)
INT8	Enabled	~500	~250	~0.16
INT8	Disabled	~1500	~1260	~0.16
FP32	Enabled	~1500	~1700	~0.19

**Table 7:** RAM and Flash Usage during Inference.

Metric	INT8	FP32
Firmware Flash (bytes)	724,469	917,453
Static RAM (bytes)	49,968	81,456
Tensor Arena Reserved (bytes)	20,147	51,635
Minimum Free Heap (bytes)	177,424	146,004

**Table 8:** Accuracy Metrics between Different Execution Environments.

Environment	Accuracy (%)	Macro-F1 (%)	Weighted-F1 (%)
Edge Impulse (FP32)	91.30	90.40	91.34
Edge Impulse (INT8)	91.34	90.36	91.40
ESP32 (FP32)	89.08	88.36	89.09
ESP32 (INT8)	90.39	89.52	90.42

**Table 9:** SONYC Evaluation Performance across Adaptation Stages (Mean ± Std, 3 Seeds).

Setting	Accuracy (%)	Macro-F1 (%)	Weighted-F1 (%)
No Adaptation	78.27%	75.63%	79.28%
ESP32_1 (1 Epoch)	82.06 ± 0.87	78.72 ± 0.61	82.96 ± 0.03
ESP32_2 (1 Epoch)	81.93 ± 0.64	78.05 ± 0.35	82.41 ± 0.41
ESP32_3 (1 Epoch)	82.90 ± 0.10	79.34 ± 0.32	82.87 ± 0.13
ESP32_4 (1 Epoch)	82.83 ± 0.50	79.20 ± 0.48	82.81 ± 0.34
ESP32_5 (1 Epoch)	80.39 ± 1.31	77.26 ± 1.32	80.98 ± 1.18
FedAvg (1 Round, 1 Epoch)	82.80 ± 0.24	79.12 ± 0.38	82.78 ± 0.18

**Table 10:** MQTT Overhead and Coordination Latency during Federated Rounds.

Metric	Measured Value
Client update payload	1.2 kB
Head collection window	180–200 ms
FedAvg aggregation time	0.17–0.19 ms
Model update latency	9–78 ms

**Table 11:** Class-Wise Recall at R0 and R30 under IID and Non-IID (α = 0.1, Mean ± Std, 3 Seeds).

Class	IID (R0)	IID (R30)	α = 0.1, N = 5 (R30)	α = 0.1, N = 50 (R30)
car_horn	52.70	83.80 ± 0.00	86.90 ± 2.55	58.10 ± 13.40
children_playing	82.80	91.10 ± 0.00	93.47 ± 0.87	91.43 ± 2.02
dog_bark	91.40	93.10 ± 0.00	88.50 ± 4.51	90.80 ± 2.16
engine_idling	62.50	90.40 ± 0.28	90.37 ± 1.11	93.87 ± 3.27
jackhammer	89.20	79.70 ± 0.00	76.13 ± 2.29	69.83 ± 2.80
siren	92.50	88.70 ± 0.00	85.80 ± 3.29	88.30 ± 0.57
street_music	87.90	55.20 ± 0.00	37.93 ± 12.51	27.60 ± 9.76

**Table 12:** ESP32 vs. Full-Model Comparison at R0 and R3 (Accuracy, Macro-F1, Class Recall; Mean ± Std, 3 Seeds).

Metric	ESP32 (R0)	ESP32 (R3)	Full-Model (R0)	Full-Model (R3)
Accuracy (%)	78.27	85.06 ± 0.09	79.62	88.78 ± 0.27
Macro-F1 (%)	75.63	82.76 ± 0.11	77.89	86.63 ± 0.18
car_horn	52.7	67.6 ± 0.9	60.8	97.3 ± 0.0
engine_idling	62.5	93.5 ± 0.8	66.8	81.7 ± 0.3
street_music	72.3	58.4 ± 0.6	82.8	78.7 ± 1.6

## Data Availability

The datasets used in this study are publicly available via Zenodo (SONYC-UST-V2, DOI: 10.5281/zenodo.3966543) and UrbanSound8K (DOI: 10.5281/zenodo.1203745). Processed datasets, label mappings, and federated partitions generated during the study are available from the corresponding author upon reasonable request.

## Abbreviations

The following abbreviations are used in this manuscript:

CNN	Convolutional Neural Network
DSP	Digital Signal Processing
FL	Federated Learning
FP32	32-bit Floating Point
HIL	Hardware-in-the-Loop
IID	Independent and Identically Distributed
IoT	Internet of Things
INT8	8-bit Integer
LPWANs	Low-Power Wide-Area Networks
MFCC	Mel-Frequency Cepstral Coefficients
MFE	Mel-Frequency Energy
MQTT	Message Queuing Telemetry Transport
MLP	Multilayer Perceptron
PDM	Pulse-Density Modulation
QoS	Quality of Service
ReLU	Rectified Linear Unit
SGD	Stochastic Gradient Descent
TFLM	TensorFlow Lite Micro
TinyML	Tiny Machine Learning
UART	Universal Asynchronous Receiver–Transmitter
